# Jujube (*Zizyphus lotus* L.): Benefits and its effects on functional and sensory properties of sponge cake

**DOI:** 10.1371/journal.pone.0227996

**Published:** 2020-02-21

**Authors:** Hanen Najjaa, Abdelkarim Ben Arfa, Walid Elfalleh, Nacim Zouari, Mohamed Neffati

**Affiliations:** 1 Laboratory of Pastoral Ecosystems and Valorization of Spontaneous Plants, Institute of Arid Regions, Medenine, Tunisia; 2 Laboratoire Energie, Eau, Environnement et Procèdes, (LEEEP) LR18ES35, Ecole Nationale d'Ingénieurs de Gabès, Université de Gabès, Gabès, Tunisia; 3 High Institute of Applied Biology of Medenine (ISBAM), University of Gabes, Medenine, Tunisia; ICAR- Indian Agricultural research Institute, INDIA

## Abstract

Jujube (*Ziziphus lotus* L.) fruit has multiple functional properties and represents an interesting source of bioactive compounds. The purpose of this study was to improve the functionality and the sensory properties of sponge cake enriched with *Z*. *lotus* fruit. The polyphenols and flavonoids levels in the sponge cake and its antioxidant potential increased with the addition of 0–10 g of jujube powder/100 g of wheat flour. The crumb color parameters, L* and b*, decreased with the addition of jujube powder, whereas the a* value increased. In the texture analysis, addition of jujube powder resulted in an increase of the hardness and chewiness, but the springiness was reduced. The sensory evaluation showed that supplementation of jujube powder did not manifest any undesirable organoleptic response and showed satisfactory consumer acceptability. Overall, the addition at 5% jujube powder showed the finest sensory properties of the sponge cake.

## Introduction

A positive relationship between foods and health has conduct to several scientific researches to find the significance of foods or food ingredients on specific body functions. The expression “functional food” refers to food with helpful functions [1]. Functional foods are considered as of the most important topics of research and innovation in the food industry [1]. Large quantities of bakery products are consumed all over the world. There are several reasons for this huge popularity including varied tastes, easy availability, extent shelf life and cheap price among other processed foods. Their evolution has experienced various stages of progress, reaching today a rising diversity [1]. Bakery products, such as cakes are so much consumed and usually, they are wealthy in carbohydrates, calories and fats, but poor in fibers, vitamins and minerals. A promising way for the food industry is to provide healthful food. Accordingly, foods enriched with dietary fibers and antioxidants have been designed, particularly in bakery products, such as cakes.

Plants are the fundamental ingredient of human food as they not only supply energy for metabolic pathways but also act as a precursor for protein synthesis, and a source of micronutrients as vitamins and minerals. *Zizyphus lotus* L. is abundant in Tunisia and popularly famous as “Sedra” and the edible fruit is called “Nbeg”. This plant is used in nutrition, health, and cosmetics in various forms (honey, juice, tea, jam, loaf, oil and cake). Besides, this plant presents a delicious red fruit (jujube) that is consumed freshly, dried and processed into flour to make pancakes with very pleasant flavor by local population [2]. The *Z*. *lotus* fruit is used also in folk medicine for the treatment of several illness, such as bronchitis, diabetes, diarrhoea, intestinal diseases and abscess [3]. The anti-inflammatory, the anticancer, the antifungal, the analgesic, the antiulcer, the antibacterial and the antispastic activities of this species were reported [4,5]. *Z*. *lotus* fruit contains important levels of carbohydrates, minerals, vitamins, fibers, amino acids, fatty acids and phenolic compounds, which are considered the main responsible for its health benefit [6].

Fortified cakes products including a significant quantity of plants and having satisfactory organoleptic attributes would be necessary to enhance the cake quality and compensate the deficit of functional and nutritional components. In this context, the current study aims to assess the effect of jujube powder substitution to wheat flour on the functional, nutritional and sensory properties of the sponge cake.

## Materials and methods

### Plant material and jujube powder preparation

Jujube fruit were obtained from wild plants, which were collected from a location near Ben Gardane (33°86′46″N, 10°25′48″E; Southeast Tunisia) in October 2017. The sampling did not involve endangered or protected species. No specific permissions were required for jujube fruit collection from this location. Indeed, the Arid Lands Institute is a public research center and is authorized by the Tunisian government conducting such research. Ben Gardane is located in arid to semi-arid region with a typical Mediterranean climate, characterized by irregular rainfall events and a harsh dry summer period. Annual precipitation is around 186 mm and annual mean temperature is 19.4°C with a minimum temperature of 3.9°C in January and a maximum of 35.9°C in August.

Jujubes with uniform shape, size, color and no defects were selected and quickly transported in open cartons to the laboratory. The plant material was washed and air-dried at room temperature (25°C). Then, all samples were sliced, and the seeds were removed before to be grounded to obtain a uniform powder stored in an airtight container for further use.

### Chemical analysis and functional characteristics of *Z*. *lotus*

The soluble dietary fibers (SDF) and insoluble dietary fibers (IDF) contents were determined according to the gravimetric enzymatic method as previously described by Prosky *et al*. [7]. Protein content (N×6.25) was estimated by the Kjeldahl digestion method followed by spectrophotometric determination of the resulting ammonia according to the method reported by Elfalleh et *al*. [8]. Total carbohydrates were evaluated by the phenol sulphuric acid method [9], after hot digestion with 1.5 M sulphuric acid. Fat content was achieved by extracting samples in a Soxhlet apparatus using petroleum ether as solvent. Moisture and ash contents were assessed according to AOAC method (2000). Water holding capacity (WHC) and fat absorption capacity (FAC) were performed by the method reported by Ayadi *et al*. [10]. WHC and FAC were expressed as g of water bound in 100 g of jujube powder and as g of oil bound in 100 g of jujube powder, respectively.

### HPLC determination of sugars

Sugars was extracted from 3 g of jujube powder using ethanol 80% solution (150 ml) for 90 min at 45°C in a Soxhlet apparatus. High-Performance Liquid Chromatography (HPLC) system Knauer instruments (Berlin, Germany) was used for sugar determination [11]. Chromatography separation was carried out on Eurospher NH_2_ column at room temperature. The acetonitrile/ultra-pure water (80%, 20%, v/v) were used as mobile phase. Sugar qualifications were determined by comparison to external standards (sucrose, fructose, and glucose). These standards were mixed to obtain a synthetic solution of 10 g/l. The peak surfaces were determined using the software Eurochrom 2000.

### Phenolic composition, LC-ESI-MS analysis and antioxidants activities of *Z*. *lotus* extract

#### Ethanol extraction

The jujube powder (1 g) was extracted by maceration using 10 ml of ethanol 70% (ethanol/water) during 2 h. The mixture was then centrifuged, and the supernatant was stored at 4°C for further analysis.

#### Total phenolic and flavonoid contents

Total polyphenol content (TPC) was determined in sample extracts using the Folin–Ciocalteu reagent ***[12]***. TPC were expressed as mg gallic acid equivalent (GAE) per gram of dry weight (mg GAE/g DW) through a calibration curve using concentrations of GA ranging from 0 to 300 mg/ml. Total flavonoids content (TFC) was estimated by colorimetric method reported by Dewanto *et al*. ***[11]***. Absorbance was measured at 520 nm and results were presented as mg of catechin equivalent (CE) per gram of dry residue (mg CE/g), using a catechin calibration curve (concentration ranging from 0 to 400 μg/ml).

#### Liquid Chromatography-electrospray Ionization-Tandem Mass Spectrometry (LC-ESI-MS) Analysis

Ethanol extract of *Z*. *lotus* fruit was filtered through a 0.45 ***μ***m filter before injection into the HPLC system. LC-ESI-MS analysis was performed using a LCMS-2020 quadruple mass spectrometer (Shimadzu, Kyoto, Japan) equipped with an electrospray ionization source (ESI) and operated in negative ionization mode according to the method reported by Jdir *et al*. [13]. Chemical standards used for the LC-ESI-MS system were HPLC grade and were purchased from Sigma Chemical Co. (St. Louis, MO, USA). All other chemicals were of analytical grade.

### Antioxidant activity of *Z*. *lotus* fruit

The scavenging ability of the DPPH^•^ (2,2-Diphenyl-1-Picrylhydrazyl)radical of *Z*. *lotus* extracts was measured according to Hanato *et al*. [14]. To 0.25 ml of DPPH^•^ methanolic solution (0.2 mmol/l) one milliliter of the extract was added. The antiradical activity was expressed as IC_50_ (μg/ml). Iron-reducing power, defined as the capacity of plant extracts to reduce Fe^3+^, was assessed by the method reported by Assadi *et al*. [11]. The absorbance was measured at 700 nm. A higher absorbance indicates a higher reducing power. EC_50_ value (μg/ml) is the effective concentration calculated from linear regression analysis with 0.5 absorbance for the reducing power.

### Sponge cake preparation

Cakes were prepared in a local pastry industry (Société Pâtisserie Masmoudi, Sfax, Tunisia: https://www.masmoudi.tn/) and the standard cake formulation consisted of 200 g wheat flour, 150 g sugar, 150 g egg, 100 g shortening, 20 g refined vegetable oil, 8 g baking powder, and 50 g milk. Cakes with variable concentrations of jujube powder were made from blends containing a mixture of wheat flour and jujube powder in the ratios of 0% (control: F1), 3% (F2), 5% (F3), and 10% (F4). The cake batter was prepared so the eggs were whipped with sugar and shortening; the flour was mixed with baking powder. The sugar–egg foam was mixed with the flour and baking powder, after which the vegetable oil was added in conforming percentage. The cake batter was poured into a silicone molds and baked at 180°C for 40 min. Cakes were cooled to room temperature. From each group (formulations F1 to F4) thirty sponge cakes were prepared to serve for further analyses.

### Color measurement

Color measurement parameters (lightness L*, redness a* and yellowness b*) were carried out using a color flex spectrocolorimeter (Hunter Associates Laboratory Inc., Reston, VA). L* value indicates the lightness, 0–100 representing dark to light, a* value gives the degree of the green–red color, with a higher positive a* value indicating more red. The b* value indicates the degree of the blue–yellow color, with a higher positive b* value indicating more yellow.

### Texture measurement

Hardness (N), springiness (mm) and chewiness (N × mm) of cakes were measured using a texturometer (Lloyd Instruments Ltd., WestSussex, UK) as previously described by Ayadi *et al*. [10].

### Sensory evaluation

The sensory properties (color, odor, taste, texture and overall acceptability) were evaluated according to the method of Murray *et al*. [15] by 60 panelists. The attributes were evaluated based on a seven-point hedonic scale, where 7: like very much, 6: like much, 5: like slightly, 4: neither like nor dislike 3: dislike slightly, 2: dislike moderately and 1: dislike very much.

### Statistical analyses

Chemical and functional characteristics of jujube powder were assessed in triplicates. All analytical determinations were performed in five replicates of sponge cake (*n* = 5) and the samples were taken randomly from five different cakes. The sensory property results were expressed as the mean of sixty replicates (60 panelists). Results were presented as Mean ± standard deviation (SD). One-way analysis of variance (ANOVA) was conducted using SPSS software, 17.0. A difference was considered statistically significant when *p*<0.05 using Duncan's Multiple Range test.

## Results and discussion

### Jujube powder characteristics: Chemical and functional characteristics

The nutritional and functional characteristics results of jujube powder were shown in **Table 1**. The table shows that carbohydrates and insoluble dietary fibers presented the most abundant constituents for jujube powder, which were higher than those of bitter melon; roselle (*Hibiscus sabdariffa*, Linn.) seeds and Osage orange (*Maclura pomifera* L.) seeds [16,17].

**Table 1:** Chemical and functional characteristics of jujube powder.

Parameters	
Moisture	6.96 ± 1.15
Ash^a^	3.65 ± 0.07
Total carbohydrates^a^	76.48 ± 0.03
Fructose^d^	13.89 ± 0.70
Glucose^d^	7.17 ± 0.41
Sucrose^d^	194.27 ± 2.44
Fat^a^	6.16 ± 0.22
Soluble dietary fibers^a^	1.70 ± 0.032
Insoluble dietary fibers^a^	19.20 ± 1.24
Proteins^a^	6.37 ± 0.33
Polyphenols^b^	412.58 ± 11.12
Flavonoids^c^	172.07 ± 24.84
DPPH^•^ scavenging activity (IC50, μg/ml)	82.00 ± 0.02
Reducing power (EC_50_, μg/ml)	36.00 ± 0.23
Water holding capacity^a^	139.00 ± 19.79
Fat absorption capacity^a^	49.00 ± 1.97

Data presented as the mean ± standard deviation (*n = 3*).

^a^: g/100 g powder. /

^b^:mg Gallic acid equivalents (GAE)/100 g powder.

^c^:mg catechin equivalents (CE)/100 g powder. /

^d^: mg/g powder.

Protein, sugars and fat amounts measured in the present study were higher compared to those reported by Boudraa [18]. This difference might occur even for the same variety of *Zizyphus*, which is mainly due to the extraction, the purification and the separation conditions [19]. *Z*. *lotu*s fruit has considerable amounts of sugars [2]. Sucrose was the most abundant in *Z*. *lotus* fruit, it was reported that other monosaccharides are frequently detected in *Z*. *lotus* fruit species, such as arabinose, galactose and rhamnose [19]. The insoluble dietary fibers content (19.20 ± 1.24 g/100g) was found to be much higher than soluble dietary fibers content (1.70± 0.032 g/100g). Dominant insoluble fraction was higher than that detected in other very consumed vegetable, such as oat, peas, beans, apples, citrus and carrots [20]. Insoluble fibers (cellulose, lignin and hemicelluloses) have an important role in the volume and intestinal transit time. Currently, consumers choose foods rich in fibers. Romo *et al*. [21] reported that the recommended quantity of dietary fibers (DF) intake per adult is of 25–38 g. Dietary fibers also possess technological characteristics that can be involved in the food’s formulation, showing in texture change and improvement of the stability of the food during production and storage. The nutritional and technological qualities of dietary fibers are interesting in the potential development of a large range of fiber-enriched foods (e.g. bakery products, snacks, sauces, drinks, cereals, biscuits, dairy products and meat products). Dietary fibers extracted from various sources have been employed to substitute wheat flour in the making of bakery products [21]. Besides, the fat amount was found to be 6.16%. The analysis of fatty acids composition of Tunisian *Z*. *lotus* fruits by Ghazghazi *et al*. [22] revealed that this oil is a main source of essential fatty acids, especially, oleic acid, elaidic acid, with more than 88% and 7% of total fatty acids, respectively. This fact offers a nutritional and medicinal potential for *Z*. *lotus* fruits. The amount of moisture was low (6.96%), which is favorable for extending the shelf life of the fruits since important moisture could cause a degradation of carbohydrates and fatty acids by microbial activity. *Z*. *lotu*s fruit has important polyphenols and flavonoids contents (**Table 1**). These values were higher than previous results described by Ghazghazi *et al*. [22] with 2.97 mg GAE/g DW of polyphenols, 1.22 mg CE/g DW of flavonoids, respectively. These variations in *Z*. *lotus* biomolecules content might be due to the environment, soil type, climate, or age of the plant. Aerial parts of *Z*. *lotus* are a potent source of polyphenols and flavonoids [23]. *Z*. *lotus* is characterized for its high amount in polyphenols revealing antioxidant, antimicrobial and immunomodulatory activities [22]. Beneficial effects of *Z*. *lotus* polyphenols on health can be generated by their antioxidant and radical scavenging properties. In addition, the obtained results of antioxidant activity showed that *Z*. *lotu*s fruits had a significant DPPH^•^ radical scavenging and reducing capacity with an IC_50_ of 82 μg/ml and EC_50_ of 36 μg/ml, respectively. IC_50_ value was found to be higher than that reported by Ghazghazi *et al*. [22] for fruit methanolic extract of the same specie (310±5 μg/ml). *Z*. *lotus* is rich in several antioxidant compounds such as phenolic acids, flavonoids and saponins. These components have been revealed to avert oxidative stress by reducing reactive oxygen species (ROS). Interestingly, numerous *in vitro* studies confirmed the capacity of *Z*. *lotus* to scavenge free radicals and to prevent cell damage [3,22]. In fact, the regular intake of natural antioxidants can reduce the risk of various diseases by reducing oxidative stress. Functional characteristics (WHC and FAC) of jujube powder were presented in **Table 1**. Hydration properties of jujube powder described by water holding capacity (WHC) showed a high value of 139±19.79 g water/100 g jujube powder that explains the ability jujube powder of a product to relate with water [24]. The high-water retention ability of jujube powder is an asset for its use to improve texture and stability of a variety of foods, such as bakery products. In addition, water absorption in the flour batter is influenced by gluten protein, damaged starch, grading, lipids, oxidizing agents and moisture content [24]. When dietary fibers is added, water absorption increases and water retention power is thereby increased; thus, dietary fibers is recognized as an aid in delaying the ageing of bakery products [25,26]. Beside the hydration capacity, jujube powder owned the ability to keep oil with fat absorption property (FAC) of 49 g fat/100 g powder that was associated principally to the surface properties of *Z*. *lotu*s fruit macromolecules. Hydrophobic constituents, such as insoluble fibers, are the main responsible of fat absorption property [27]. This property could be used in some foods to enhance their retention of fat and flavor and to increase the technological yield.

### Liquid chromatography-electrospray ionization-tandem mass spectrometry (LC-ESI-MS) analysis of *Z*. *lotus* fruit extract

LC-ESI-MS analysis of *Z*. *lotus* fruit extracts identified 14 phenolic compounds that were classified into 7 phenolic acids and 7 flavonoids (**Table 2**). The compounds identification was performed by comparing retention times and mass spectra with those of the authentic standards. Phenolic compounds including quinic acid, rutin, gallic acid, protocatechic acid and *trans*-cinnamic acid were quantified in *Z*. *lotus* fruit (**Table 2**). In fact, some compounds such as gallic acid, syringic acid, catechin, quercetin, rutin and kaempferol were previously reported in *Z*. *lotus* fruit [28]. An overview of the literature indicates that certain of these identified compounds had powerful antioxidant capacity with IC_50_ values less than 10 μg/ml except coumaric acid, quercetin and naringenin (**Table 2**). The quinic acid is the major compound (2769.41 μg/g extract) in *Z*. *lotus* fruit extract followed by rutin (808.26 μg/g) and gallic acid (110.10 μg/g). These three compounds seem effective antioxidant potency with respective IC_50_ of 2.6, 9.6 and 3.5 μg/ml. Therefore, the consumption of food products enrichment with *Z*. *lotus* fruit would potentially supply antioxidant potential and thus health benefits.

**Table 2:** LC-ESI-MS analysis of the *Z*. *lotus* fruit extract and literature review of the DPPH^•^ radical-scavenging presented as extract concentration needed to scavenge 50% of DPPH^•^ (IC_50_) values.

Compounds	Formula	MW	[M-H]- m/z	Rt (min)	Content (μg/g)	IC_50_(μg/ml)	Reference
Quinic acid	C_7_H_12_O_6_	192	191	2.161	2769.41	2.60	Brighente*et al*. [39]
Gallic acid	C_7_H_6_O_6_	170	169	3.946	110.10	3.53	Singh *et al*., [40]
Protocatechuic acid	C_7_H_6_O_4_	154	153	6.903	62.94	0.89	Kakkar *et al*., [41]
Catechin (+)	C_15_H_14_O_6_	290	289	11.086	7.16	6.38	Hsu *et al*., [42]
Syringic acid	C_9_H_10_O_5_	198	197	16.029	6.75	0.50	Dias *et al*.[43]
p-Coumaric acid	C_9_H_8_O_3_	164	163	20.895	66.76	105.3	Ayranci *et al*.[44]
*trans*-Ferulic acid	C_10_H_10_O_4_	194	193	23.100	8.97	3.34	Mishra *et al*., [45]
Hyperoside	C_21_H_20_O_12_	464	463	24.610	5.40	5.19	Zhao *et al*., [46]
Rutin	C_27_H_30_O_18_	610	609	23.874	808.26	9.60	Tang *et al*., [47]
Rosmarinic acid	C_18_H_16_O_8_	360	359	26.904	0.48	0.80	Kindl *et al*., [48]
*trans*—Cinnamic acid	C_9_H_8_O_2_	148	147	31.789	62.68	-	-
Quercetin	C_15_H_10_O_7_	302	301	31.913	5.99	10.57	Khanduja *et al*., [49]
Naringenin	C_15_H_12_O_5_	272	271	33.794	6.46	18.00	Brighente *et al*., [39]
Cirsiliol	C_17_H_14_O_7_	344	329	35.462	7.49	2.34	Yokozawa *et al*., [50]

### Enriched cake quality

#### Phenolic and flavonoids contents and antioxidant activity of the jujube enriched cake

**Table 3** shows the results of polyphenols and flavonoids contents and the percentages of inhibition of DPPH^•^ free radical for diverse cake formulations. Our study is the first report on sponge cake prepared using jujube powder. The tendency of polyphenols and flavonoids showed a positive association with jujube powder substitution into the cake formulation. This is in conformity with the findings of Uthumporn *et al*.[29]. Formulation F4 showed the uppermost TPC value compared to control cakes and the uppermost inhibition of DPPH^•^ free radical capacity. The higher incorporation of jujube powder into cakes had increased the antioxidant activity for few folds compared to the control cakes without jujube powder (Control < F2 < F3 < F4). This correlation was principally attributed to the fact that jujube contains an important content of phytochemicals notably phenolic compounds exhibiting high antioxidant activity [22]. This result is in accordance with Uthumporn *et al*. [29], who reported that cakes with eggplant flour substitution provided better antioxidant capacities with a linear relation between the TPC and antioxidant properties. The flavonoids and phenolic acids identified in *Z*. *lotus* fruit (**Table 2**) can participate individually or synergistically to the antioxidant capacity indicated in the cake enriched with the jujube powder. Comparable studies illustrated that the fortification of cakes with natural raw materials, such as mallow and sour cherry pomace extract enhanced its antioxidant capacity and a reduction in oxidative decomposition during the storage of supplemented cakes [30,31].

**Table 3:** Polyphenols, flavonoids and antioxidant activity of cake enriched with jujube powder.

Substitution level (g/100 g cake)	Polyphenols	Flavonoids	Scavenging activity
F1	9.89 ± 0.04^d^	4.01 ±0.03^d^	5.29 ± 0.08^d^
F2	18.85 ± 0.05^c^	10.89 ± 0.02^c^	23.04 ± 0.05^c^
F3	29.25 ± 0.04^b^	16.44 ± 0.40^b^	31.01 ± 0.04^b^
F4	38.12 ± 0.02^a^	23.13 ± 0.04^a^	54.02 ± 0.05^a^

Each value in the table is represented as mean ± SD (*n* = 5).

^a, b, c, d^ Values with the same superscript letters in the same line are non-significant at *p*<0.05; polyphenols are expressed as mg gallic acid equivalents (GAE)/100 g cake; flavonoids are expressed as mg catechin equivalents (CE)/100 g cake; DPPH^•^ scavenging activity (%) is determined at 0.1g/ml cake extract.

Therefore, the antioxidant potential of jujube-supplemented cakes improved their nutritional quality with better stability against oxidation. Thus, cake enrichment with jujube appears to be a very facile and low-cost way for food processing and quality enhancement.

#### Instrumental texture, and color properties

**Table 4** shows that there was significant difference (*p*< 0.05) between the hardness value of control cakes and the other cakes substituted with jujube powder. Cakes prepared with jujube have the hardest texture (5.33±0.01, 7.51±0.05 and 7.81±0.45 for 3%, 5% and 10%, respectively) compared to the control cakes. The hardness of cakes is highly affected by the composition of flour. Some studies revealed that there was positive relationship of fibers and protein levels with the hardness value of cakes prepared [32].

**Table 4:** Effect of jujube powder on the cake texture.

Substitution level (g/100 g wheat flour)	Hardness (N)	Springiness (mm)	Chewiness (N× mm)
F1	8.09 ± 0.05^a^	9.85 ± 0.21^a^	8.09 ± 0.05^a^
F2	7.29 ± 0.10^b^	11.67 ± 0.45^b^	7.29 ± 0.10^b^
F3	6.24 ± 0.13^c^	19.85 ± 0.58^c^	6.24 ± 0.13^c^
F4	6.01 ± 0.01^d^	20.63 ± 0.70^d^	6.01 ± 0.01^d^

Each value in the table is represented as mean ± SD *(n* = 5).

^a,b,c,d^ Values with same superscript letters in the same row are non-significant at *p*< 0.05.

The raise of cakes hardness is mainly attributed to the amount of fibers added. According to Noda *et al*. [33], dough made from high-absorption flour will have a hard texture. **Table 4** also shows that cakes enrichment with jujube powder resulted in a significant increase (*p*<0.05) of chewiness of cake but a significant decrease (*p*<0.05) of its springiness. Color is one of the most principal characteristics of foods, being supposed as a quality key that defines their acceptance. **Table 5** shows that control cakes had a significant difference in terms of L*, a*, and b* values compared to all other cakes prepared by substitution with jujube powder. The control cake offered significantly (*p*<0.05) highest L* value compared to *Z*. *lotus* substituted cakes (56.52±3.18%). A decease in L* was illustrated with the substitution of jujube powder (56.52 in control to 46.37 and 43.10 in cakes made with 5 and 10% of jujube powder level, respectively). This statement was confirmed by Vratanina and Zabik [34], who supported that the reduction in lightness of cake was noted as the addition amount of fibers into formulation was increased. The significant difference (*p*<0.05) in a* and b* values detected in control cakes compared to other cakes could be due to various exposure of cake surface to high baking temperature. Consequently, colored compounds were generated from caramelization and Maillard reaction which occurred during baking [35]. Color tonality of cakes is principally depending on the level of sugars and proteins in the formulation because Maillard reaction is the first chemical reaction in the bakery products during baking. The level of water on the dough surface dramatically reduced when heat was applied, which provided a best condition for Maillard reaction to occur in product and showed in strong brown color. Sudha *et al*. [36] investigated the effect of fibers substitution from several cereals on the biscuit quality. These authors announced that the biscuits became darker with augmentation amount of either of the bran except for barley bran addition where the percent whiteness was decreased marginally. In the case of adding a powder different from flour, browning is influenced by the type and color of the added powder, in addition to the folding process in confectioneries and the baking of products [36,37]. At the same time, cakes color values raised as jujube powder incorporation level raised. L*values changed from white to gray, a* values changed from green to red and b* values changed from blue to yellow. Then, increasing the amount of jujube from 3 to 10% decreased L*and b* values and increased a* values (redness). This redness in samples with jujube powder (significantly higher in F3 and F4 than in F2 and F1) attribute to the presence of anthocyanins. These results are in accordance with the results of Kim *et al*. [38] who also confirmed that lightness (L*) and yellowness (b*) of cakes’ color decreased as the level of cherry powder increased, while the redness (a*) increased.

**Table 5:** Color characteristics of crust of the cake enriched with jujube powder.

Substitution level (g/100 g cake)	L*	a*	b*
F1	56.52 ± 3.18^a^	-0.54 ± 0.37^a^	22.49 ± 1.71^a^
F2	50.11 ± 0.72^b^	2.51 ± 1.14^b^	16.78 ± 0.50^b^
F3	46.37 ± 0.79^b^^c^	4.27 ± 0.53^c^	14.53 ± 0.77^c^
F4	43.10 ± 0.48^c^	6.63 ± 0.09^d^	8.55 ± 0.13^d^

Each value in the table is represented as mean ± SD (*n* = 5).

^a,b,c,d^ Values with same superscript letters in the same row are non-significant at *p*< 0.05.

L*, a* and b* represent bright, red and yellow indexes, respectively.

#### Sensory evaluation of cakes

**Fig 1:**
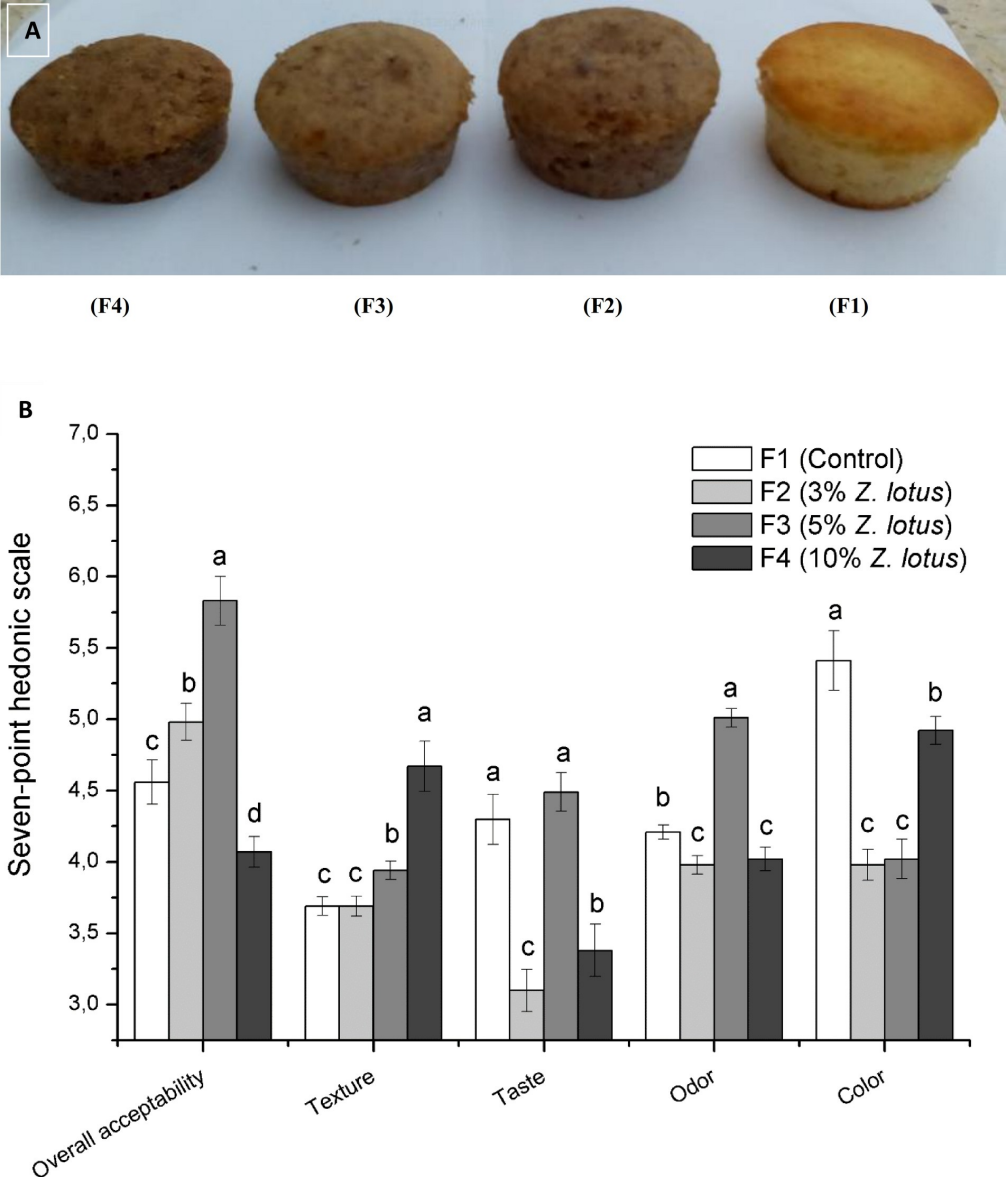
**(A) Cake prepared with 3% (F2), 5% (F3) and 10% (F4) of *Z*. *lotus* fruit powder. (B) Sensory evaluation of the formulated cakes.** The control (F1) represented the product without enrichment. The attributes were evaluated using a seven-point hedonic scale, where 7: like very much, 6: like much, 5: like slightly, 4: neither like nor dislike 3: dislike slightly, 2: dislike moderately and 1: dislike very much. Each value is represented as mean ± SD (n = 60). Values with same superscript letters in the same attribute are non-significant at *p*< 0.05.

The sensory quality of cakes was evaluated through taste, surface color and texture to determine the products acceptability score. **Fig 1** reveals how organoleptic qualities of assessed cakes were affected by adding 3%, 5%, and 10% of jujube powder. **Fig 1A** also shows photos of cakes with jujube powder substitution. **Fig 1B** reveals a change in cakes quality with enrichment of jujube powder.

In fact, with the increase of jujube substitution, cakes crust became darker, especially at 5 and 10%. Likewise, surface cake downs were also observed with the increased of jujube powder addition. Panelist awarded more scores for texture, odor, taste and overall acceptability of fresh cakes at 5%, whereas color scores were observed more at 10% level of jujube powder. Oppositely, consumer scores were reduced with increasing supplementation level of cladodes and pomegranate peel powder in the formulation of cakes [3]. Surface color of cakes is an important factor to determine its acceptability. Jujube powder gave brown color characteristic to the final product (**Fig 1**). The highest color scores attributed to the cake made with jujube (F4 (10%)) followed by cakes with 5 and 3% jujube powder supplementation) because it showed a nature color of chocolate. Concerning the taste, higher likeness was attributed by the panelist at 5% supplementation level and the control. The taste of cakes was sweet at 5% level of substitution. Jujube is characterized by good taste and pleasant flavor, therefore, was evident to produce cakes with higher acceptable taste and flavor properties.

In terms of Overall acceptability rating, cake at 5% *Z*. *lotus* gave the highest consumer acceptability. It was concluded then, that jujube powder could be incorporated up to 5% in the formulation of cakes since the obtained mean score for the overall acceptability was 5.8; while all cake containing 3 and 10% concentration of jujube powder (F2 and F4) kept acceptable since the obtained mean score for the overall acceptability were 4.8 and 4.6, respectively, on a 7-point hedonic scale.

## Conclusion

With a wide ecological and geographical distribution in Tunisia and other Mediterranean countries and grows under a variety of environmental conditions, *Z*. *lotus* plant grows wild and consumed as fresh fruit. *Z*. *lotus* fruits could be a good dietary supplement with high bioactive compounds contents, such as dietary fibers, mineral, and natural antioxidant compounds. A successful and novel formulation for sponge cake production using jujube powder was developed. Sponge cakes containing a partial replacement of cake flour with up to 10 g/100 g sample had bioactive compounds and a pleasant texture and taste compared with cake prepared only wheat cake flour. The use of jujube powder in sponge cake is deemed to be good. Overall, jujube powder can be incorporated into cake, providing more functional components and a more potential health benefits to the consumers.

## Supporting information

S1 Fig Graphical abstract. (DOCX)

S1 Table All raw data are available in the supplementary (xls) Tables. (XLS)
