# Supplementary material for: Jujube (Zizyphus lotus L.): Benefits and its effects on functional and sensory properties of sponge cake
Source: PLoS One. 2020 Feb 21;15(2):e0227996. doi: 10.1371/journal.pone.0227996 (PMC7034905; doi:10.1371/journal.pone.0227996)
Supplement: S1 Fig — (DOCX) [file pone.0227996.s001.docx]

**Graphical abstract**


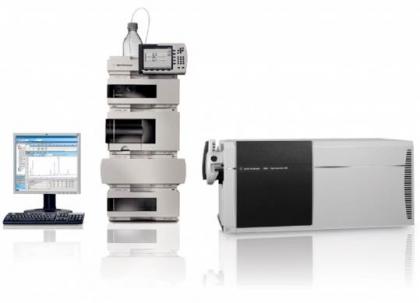


**Bioactive compounds: Flavonoids, phenolics acids**


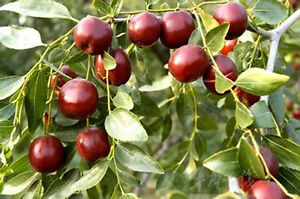


**Improvement of antioxidant and** **physical, sensory properties**

**Jujube**

***(Zizyphus Lotus*)**

**Wheat flour**

**Substitution**


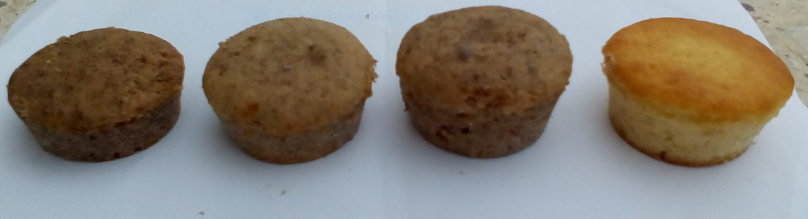


(T) (3%) (5%) (10%)

**Cake making**
